# Comparison of oxyhemoglobin and deoxyhemoglobin signal reliability with and without global mean removal for digit manipulation motor tasks

**DOI:** 10.1117/1.NPh.5.1.011006

**Published:** 2017-09-14

**Authors:** Swethasri Dravida, Jack Adam Noah, Xian Zhang, Joy Hirsch

**Affiliations:** a Yale School of Medicine, Interdepartmental Neuroscience Program, New Haven, Connecticut, United States; b Yale School of Medicine, Department of Psychiatry, New Haven, Connecticut, United States; c Yale School of Medicine, Department of Neuroscience, New Haven, Connecticut, United States; d Yale School of Medicine, Department of Comparative Medicine, New Haven, Connecticut, United States; e University College London, Department of Medical Physics and Biomedical Engineering, London, United Kingdom

**Keywords:** reliability, functional near-infrared spectroscopy, finger-tapping, global mean

## Abstract

Functional near-infrared spectroscopy (fNIRS) could be well suited for clinical use, such as measuring neural activity before and after treatment; however, reliability and specificity of fNIRS signals must be ensured so that differences can be attributed to the intervention. This study compared the test–retest and longitudinal reliability of oxyhemoglobin and deoxyhemoglobin signals before and after spatial filtering. In the test–retest experiment, 14 participants were scanned on 2 days while performing four right-handed digit-manipulation tasks. Group results revealed greater test–retest reliability for oxyhemoglobin than deoxyhemoglobin signals and greater spatial specificity for the deoxyhemoglobin signals. To further characterize reliability, a longitudinal experiment was conducted in which two participants repeated the same motor tasks for 10 days. Beta values from the two tasks with the lowest and highest test–retest reliability, respectively, in the spatially filtered deoxyhemoglobin signal are reported as representative findings. Both test–retest and longitudinal methods confirmed that task and signal type influence reliability. Oxyhemoglobin signals were more reliable overall than deoxyhemoglobin, and removal of the global mean reduced reliability of both signals. Findings are consistent with the suggestion that systemic components most prevalent in the oxyhemoglobin signal may inflate reliability relative to the deoxyhemoglobin signal, which is less influenced by systemic factors.

## 1 Introduction

Functional near-infrared spectroscopy (fNIRS) is a neuroimaging technique that records changes in blood oxygen levels, which are used as a proxy for localized neural activity. Recent advances in fNIRS hardware allow for whole-brain imaging in ecologically valid contexts and have prompted a dramatic increase in fNIRS research.^1^ Of particular interest are longitudinal studies, including studies that focus on changes in brain activity underlying learning and training.^2^^–^^4^ fNIRS is a relatively inexpensive, radiation-free method of obtaining continuous or repeated measurements that can be used to evaluate learning, training, intervention, or neurofeedback. For these applications, it is necessary to understand the reliability of the NIRS signal with respect to changes in oxyhemoglobin (OxyHb), deoxyhemoglobin (deOxyHb), and systemic effects, particularly when signal changes may reflect either the outcome of a treatment or signal attenuation.

Reliability is defined as the reproducibility of a measurement.^5^ Variation in the reliability of neural recordings can come from many sources, including equipment, signal-to-noise ratio, and participant variability. Test–retest reliability is an indicator of consistency in repeated measurements made with one particular method or tool.^6^ A number of reports have previously investigated test–retest reliability using fNIRS. Some of these reports compared reliability in blood oxygen saturation across devices.^7^ In one study, Yoshitani et al. showed that the type of NIRS machine and the methodology used to obtain signals can influence the measures of blood oxygen saturation (${\mathrm{SO}}_{2}$) differentially in the presence of changing ${\mathrm{CO}}_{2}$ concentration in the blood. A number of methodological issues were reported for discrepancies, including how blood saturation was measured by each machine, the influence of extracranial blood flow on both measures, and the source of light used for measurements (laser diode versus LED). Reliability in tissue saturation in infants^8^ has also been investigated. This study reported test–retest and inter-rater reliability for changes in hemoglobin measures as well as oxygen saturation using NIRS on infants during resting state. The results showed that ${\mathrm{SO}}_{2}$ measures were reliable both day to day as well as between raters, but significant differences in hemodynamic measures were found between baseline measures and across raters. Errors in placement of the single measurement channel were one potential source of error suggested by the authors. Functional NIRS has also been evaluated for reliability with respect to its use in cognitive screening.^9^ This study showed mixed results for the reliability of OxyHb and deOxyHb in multiple areas of the frontal lobe but suggested OxyHb may be more reliable for some types of cognitive tasks. Visual and auditory stimulation,^10^ as well as motor output tasks,^11^ have also been tested for reliability. In the 2006 paper, the authors showed that OxyHb signals during a passive visual viewing task were more reliable than deOxyHb. In their 2007 study, they found that deOxyHb showed more localized responses in a finger-thumb tapping task, but reliability was low for both oxy and deOxyHb signals. The authors suggest that differences in probe placement may have contributed to some of the variability. While there is general agreement about basic mechanisms of action relating to tissue saturation and changes in hemoglobin concentration during functional activity, the relative reliability of OxyHb and deOxyHb remains an active area of research. The goal of this experiment was to build on what has been previously reported and evaluate the effects of global mean removal on the reliability of OxyHb and deOxyHb signals.

Functional NIRS records changes in oxyhemoglobin and deoxyhemoglobin concentrations. The specific changes in hemodynamic signals recorded with fNIRS reflect underlying neural activity but may also contain multiple sources of systemic effects.^12^^–^^14^ Two techniques are commonly used for removing systemic components from fNIRS signals. The first is short channel separation, which has been shown to be able to remove a localized artifact that is non-hemodynamic in its temporal activation profile; however, removal of artifacts that are very similar in temporal response to neural activity, such as changes in blood pressure,^15^ may also regress out true neural responses. The second technique is a principle components analysis (PCA) spatial filter^14^ that removes activity distributed across the entire cortex, which has been shown to be effective in isolating neural responses. We evaluate this spatial filter to determine how systemic components affect the reliability of fNIRS recordings.

Here, we investigate the effects of signal and task type on the reliability of fNIRS data. The overall goal of this study was to compare the reliability of fNIRS signals in the motor cortex in a test–retest experiment. Specifically, we obtained whole-head fNIRS recordings during a series of digit manipulation tasks in which participants perform stress ball squeezing, finger-thumb tapping, double finger-thumb tapping, and a finger-thumb tapping task in which participants tapped specific digits against their thumb when cued by a number. Expected responses in the contralateral motor cortex were observed for all tasks across participants on day one and day two. We compared reliability from day one to day two in OxyHb and deOxyHb signals in all tasks both before and after spatial filtering of systemic components. We specifically assessed whether OxyHb or deOxyHb was a more reliable signal for each of the four motor tasks. Finally, we determined how the OxyHb and deOxyHb signals varied in a longitudinal experiment in which two participants repeated the same four motor tasks for 10 days.

## 2 Test–Retest Experiment Methods

### 2.1 Participants

Fourteen participants (4 male, 10 female; mean age: 26.9+/−9.5 years; 100% right-handed ^16^) took part in the experiment over 2 days. Participants provided written informed consent in accordance with guidelines approved by the Yale University Human Investigation Committee (HIC #1501015178). All data were obtained at the Yale School of Medicine, New Haven, Connecticut. Each person was compensated for participation in the study.

### 2.2 Paradigm

In the test–retest experiments, participants completed four tasks that required right-handed digit manipulation. For the first task (“ball squeeze”), participants squeezed an elastic stress ball in response to cues presented on a computer screen. During the second task (“double finger tap”), participants tapped each finger sequentially against the thumb twice per cue. For the third run (“finger tap”), participants tapped each finger sequentially against the thumb once per cue. During the fourth run (“follow the number”), a number from 1 through 4 appeared randomly on the screen. Participants were instructed to tap the first finger against the thumb in response to “1,” the middle finger in response to “2,” the ring finger in response to “3,” and the pinky finger in response to “4.” Each run consisted of six blocks. Each block consisted of 20 s of task followed by 10 s of rest, during which participants were instructed to focus on a crosshair on the screen and keep their hands still. There were 24 cues presented every 0.83 s during the 20-s task block.

### 2.3 Signal Acquisition

**Fig. 1:**
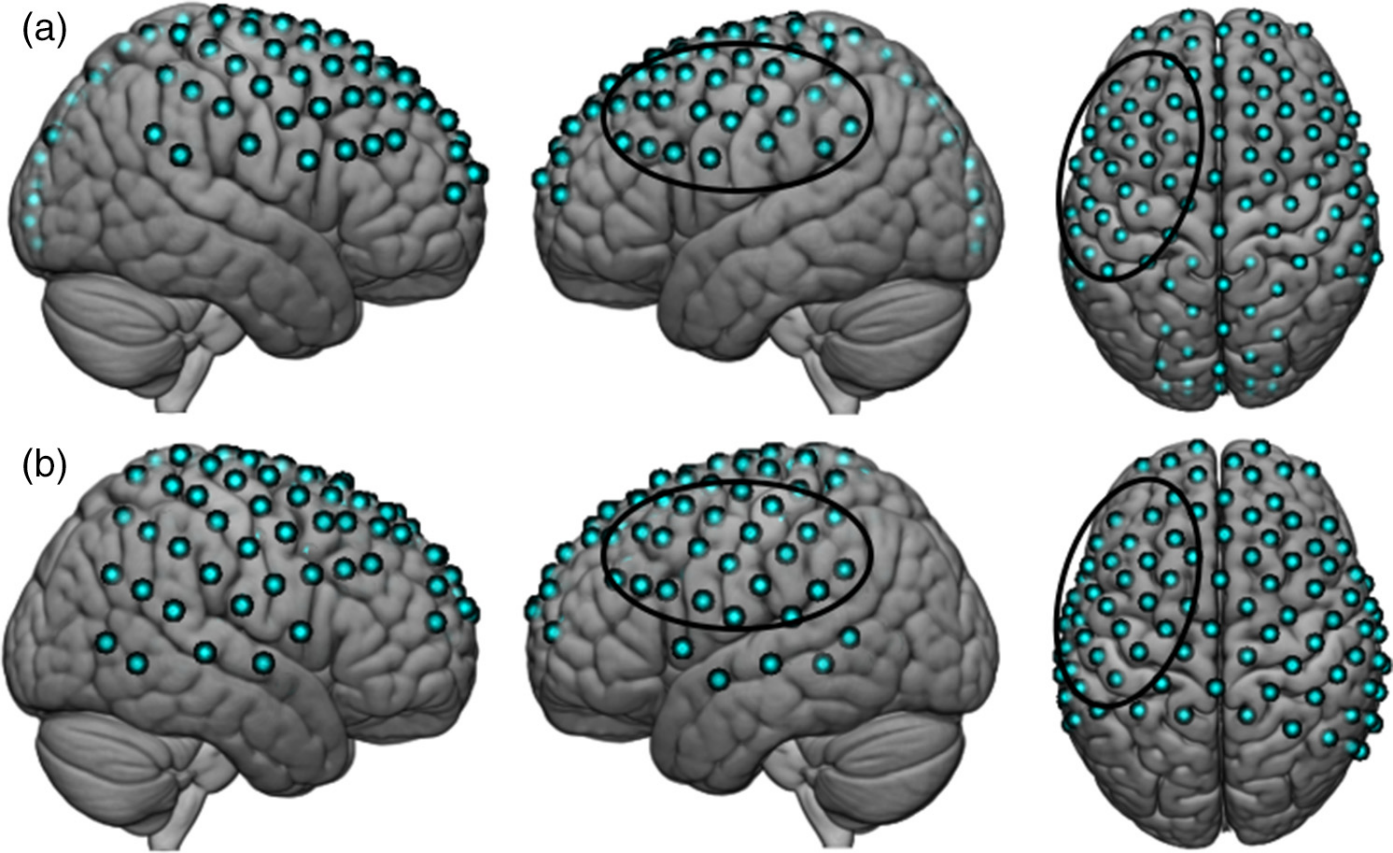
Channel layouts. (a) For the test–retest experiment, 32 detectors and emitters were arranged in a 105 channel layout, represented by the blue circles. This layout covered the frontal, temporal, parietal, and occipital lobes. Black ovals surround 29 channels used for the ROI. (b) For the longitudinal experiment, 32 detectors and 29 emitters were arranged in a 98 channel layout. Black ovals surround 23 channels used for the ROI.

Data were acquired using a multichannel, continuous wave Shimadzu LABNIRS system (LABNIRS, Shimadzu Corp., Kyoto, Japan), which consists of emitters that connect to laser diodes at three wavelengths (780, 805, and 830 nm). Each participant was fitted with an optode cap with predefined distances of 2.75 or 3 cm depending on the size of the individual’s head. The cap was placed so that the most anterior optode-holder was positioned ∼1  cm above nasion and the most posterior opode holder 1 cm below inion. These anatomical landmarks were chosen to maximize the chance that the cap was placed on a single participant’s head the same way each day. Hair was removed from the channel area prior to placement of each optode using a lighted fiber optic probe (Daiso, Hiroshima, Japan). Thirty-two emitters and detectors were arranged in a 105-channel layout covering the full head [Fig. 1(a)]. The resistance in each channel was measured prior to recording and adjustments were made until the channel resistance met the minimum LABNIRS requirements.^4^^,^^17^^,^^18^ Signals were down-sampled 10-fold during the analysis for an effective sample rate of 1.0 s.

### 2.4 Optode Localization

Following signal acquisition, the optodes were removed from the cap, but the cap was left on the participant for the purpose of optode localization. Anatomical locations of optodes with respect to the standard 10 to 20 system^19^ head landmarks nasion, inion, Cz, T3 (left tragus), and T4 (right tragus) were determined using a Patriot 3-D Digitizer (Polhemus, Colchester, Vermont) and previously described linear transform techniques.^20^^–^^23^ The NIRS-SPM software^24^ was used with MATLAB (Mathworks, Natick, Massachusetts) to determine Montreal Neurological Institute (MNI) coordinates for each channel. The corresponding anatomical locations for each channel were determined using the Talairach atlas.^25^^,^^26^

### 2.5 Signal Processing

A modified Beer–Lambert equation was used to convert raw fNIRS data to deoxyhemoglobin and oxyhemoglobin concentrations, and wavelet detrending was applied to these values. A fourth-degree polynomial was used to model and remove the baseline drift from the raw signal. For each participant, channels were automatically removed from the analysis if the root mean square of the raw data trace was 10 times that of the average for that participant. Comparisons between “clean” and “raw” data refer to data that did or did not undergo global mean removal, respectively. To generate the “clean” data, global systemic effects were removed using a spatial filter^14^ prior to hemodynamic modeling. The assumption underlying the use of a spatial filter is that neural activity due to the task, in this case related to finger movements, would result in activity localized to the contralateral motor cortex. Therefore, any activity present across a larger area of the brain is most likely due to global systemic effects. The algorithm used here^14^ utilizes PCA and a high-pass Gaussian spatial filter to remove components of the data that are present throughout the brain. Raw and clean data were reshaped into 4×4×4×133 images, and SPM8 was used for first-level general linear model (GLM) analysis.

### 2.6 Contrast Comparisons

The GLM for fNIRS was used to generate contrast comparisons for each task versus rest.^27^ The 30-s experimental blocks, which included the 20-s task blocks and 10 s of rest, were convolved with the hemodynamic response function and modeled to fit the data. This resulted in individual beta values for each participant for every task. Beta values were obtained for all channels. One-tailed t-tests were used to generate group-level data in SPM8. Results were rendered at a threshold of p<0.005.

### 2.7 Test–Retest Reliability

To evaluate the reliability of activity in the motor cortex from day 1 to day 2, each participant’s channel locations were converted to MNI space. Each participant’s data were then registered to the median channel location of both days using a nonlinear interpolation method. Once in normalized space, registered beta values were used to calculate test–retest reliability over 2 days. Beta values in all channels from all four tasks from both days were averaged, and the channel with the maximum beta value in a preidentified region of interest (ROI) was identified for each participant [black ovals in Fig. 1(a)]. The ROI comprised 29 channels in the left hemisphere, covering premotor, primary-motor, and supplementary motor areas. The channel with the maximum average beta value, the channel of interest, differed across participants. Once the channel of interest was identified, beta values in that channel were extracted for each task from both days for each participant. The intraclass correlation coefficient (ICC) was used to compare the degree of reliability between the beta values on day 1 versus day 2. A MATLAB script was used to generate the ICCs for each signal type (deOxyHb and OxyHb) and each processing type (raw and clean) for each digit manipulation task.

### 2.8 Similarity of Day-to-Day Cap Placement

The reliability of the cap placement from day to day was confirmed by taking the channel of interest for each participant and calculating the distance between the MNI coordinates for this channel on day 1 and the MNI coordinates for the same channel on day 2. The average distance between the channel of interest on day 1 and day 2 was 9.5±6.7  mm, confirming that variations in cap placements on both days were within the spatial resolution of 3 cm.

In this study, the single channel with the maximum beta value in the ROI was used to identify beta values as the measure of reliability because data from a single channel reflect the most specific local neural activity from each participant. This was intended to eliminate variability in the group location of activity due to variation in head shape across individuals. Variations in the location of the channel of interest for a single subject between day 1 and day 2 were within the spatial resolution of a single channel and, therefore, contributions to measures of reliability were not detectable. Within an individual subject, neural activity related to the task was restricted to a few channels in the primary motor or premotor/supplementary motor cortex with peak activity in one. Only in the group results, when the data were interpolated across subjects, did the combined activity cover a larger area of cortex. Using an average of the beta values in the entire area in a single individual would have therefore resulted in averaging “zeros” from channels with no significant activity, reducing the chance of comparing real activity from day to day.

## 3 Test–Retest Results

### 3.1 Contrast Results

**Fig. 2:**
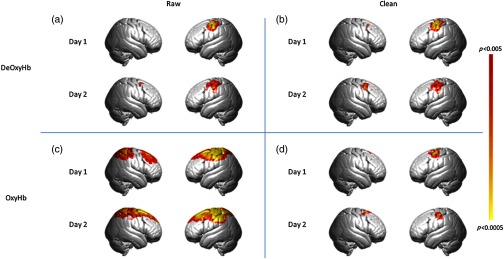
Results of all tasks contrast, p<0.005. The top row represents the group-averaged deOxyHb data; bottom row represents OxyHb data. (a) Results of raw deOxyHb data from day 1 and day 2. (b) Results of clean (spatial filter applied) deOxyHb data from day 1 and day 2. (c) Results of raw OxyHb data from day 1 and day 2. (d) Results of clean (spatial filter applied) OxyHb data from day 1 and day 2.

The group-level results for all combined tasks versus the rest in the test–retest experiment are shown in Fig. 2 and areas in the left motor regions are reported in the tables in the Appendix. The results of the group-level contrast from each individual task are also shown in the Appendix. As predicted, each of the right-handed digit manipulation tasks resulted in activity in the left premotor, primary motor, and supplementary motor cortices. Results are presented for raw and clean data (Fig. 2, columns) for each signal type (OxyHb and deOxyHb, Fig. 2, rows). Consistent with prior studies,^13^^–^^15^ raw OxyHb data showed a distributed pattern of activity that became localized when the spatial filter was used, while deOxyHb signals were localized to motor cortex for both raw and clean results.

### 3.2 Test–Retest Signal Reliability

ICCs were used as a measure of test–retest signal reliability. The ICC values were determined for each task and both types of signal: deOxyHb versus OxyHb with and without global mean removal. The overall ICC value for each signal type was also calculated using the beta values from all tasks from both days. The ICCs are shown in Table 1. When collapsed across raw and clean data, the ICC values for the four tasks obtained from the OxyHb signal were significantly greater than those obtained from the deOxyHb signal (one-tailed paired t-test, p=0.048). When collapsed across OxyHb and deOxyHb data, the ICC values for the four tasks using the raw data were significantly greater than the ICC values for the clean data (one-tailed paired t-test, p=0.0086).

**Table 1:** Test–retest ICC for each task and for the combination of all tasks.

	DeoxyHb		OxyHb	
	Raw	Clean	Raw	Clean
Ball squeeze	0.5906	0.3851	0.8194	0.7086
Double tap	0.5922	0.4539	0.8463	0.8307
Single tap	0.6815	0.5687	0.7374	0.5142
Follow the number	0.6580	0.6722	0.5878	0.5843
**All tasks**	**0.6188**	**0.5020**	**0.7659**	**0.6588**

## 4 Longitudinal Experiment

We compared the amount of systemic component versus neural signals in these tasks by conducting a second experiment (referred to as the longitudinal experiment) in which two participants performed the same four tasks every day for 10 days. The basis for this experiment was the expectation that when a participant undergoes the same task every day, neural signals will attenuate over time due to a repetition effect.^28^^–^^30^ The hypothesis for this experiment was twofold. First, we predicted that tasks that generate more systemic artifacts would show less attenuation in the OxyHb data over the course of the 10 days than tasks that generate fewer artifacts. Second, we hypothesized that deOxyHb data would show similar attenuation in all tasks, as this signal is less affected by systemic artifacts.^12^^,^^14^^,^^15^

## 5 Longitudinal Experiment Methods

### 5.1 Participants

Two right-handed participants (one 25-year-old female and one 42-year-old male) participated in the longitudinal experiment and were tested for 10 days. As above, participants provided written informed consent in accordance with guidelines approved by the Yale University Human Investigation Committee (HIC #1501015178), and all data were obtained at the Yale School of Medicine, New Haven, Connecticut.

The task paradigms, signal acquisition, and signal processing methods used were the same as in the test–retest experiment (see Secs. 2.2–2.6 above). For this experiment, 32 emitters and 29 detectors were arranged in a 98-channel layout [Fig. 1(b)], as coverage of the occipital lobe was deemed unnecessary. The ROI consisted of 23 channels in the left hemisphere [black ovals in Fig. 1(b)].

### 5.2 Intraparticipant Signal Consistency

The same channel registration method described in the first experiment was used to register the data from all 10 days to one set of channel locations for each participant. To evaluate interscan variability, registered beta values from each channel were averaged over 10 days for each participant. The channel with the maximum average beta value in the left motor cortex ROI was identified for each participant, and beta values in this channel were identified for every task performed over 10 days for each participant using raw and clean signal data. As with the test–retest experiment, the channel of interest was selected for each participant. Main effects from the ball squeeze and follow the number tasks are shown below. These two tasks were chosen because they showed the greatest difference in test–retest reliability in the clean, deOxyHb signal. Out of the four tasks, the ball squeeze task showed the lowest reliability (ICC=0.3567) and the follow-the-number task showed the highest reliability (ICC=0.6264) using the deOxyHb signal with the spatial filter, which was consistent with the least amount of systemic artifact. We compared the longitudinal beta values using the deOxyHb and OxyHb signals with and without the spatial filter from both of these tasks for each participant over the course of 10 days.

### 5.3 Regression Slope Tests

The beta values in the channel of interest were plotted over the 10 days for each participant in each task. A trend line was fitted to the data, and a regression slope test was performed on each trend line, to evaluate whether the slope was significantly different from zero. In a regression slope test, a t statistic is obtained by dividing the slope of the line by the standard error of the slope. This t statistic was then converted to a p value using the degrees of freedom (number of points -2). A p value less than 0.05 was considered to be significant, namely that the slope of the trend line over the 10 days was significantly different from zero.

## 6 Longitudinal Experiment Results

**Fig. 3:**
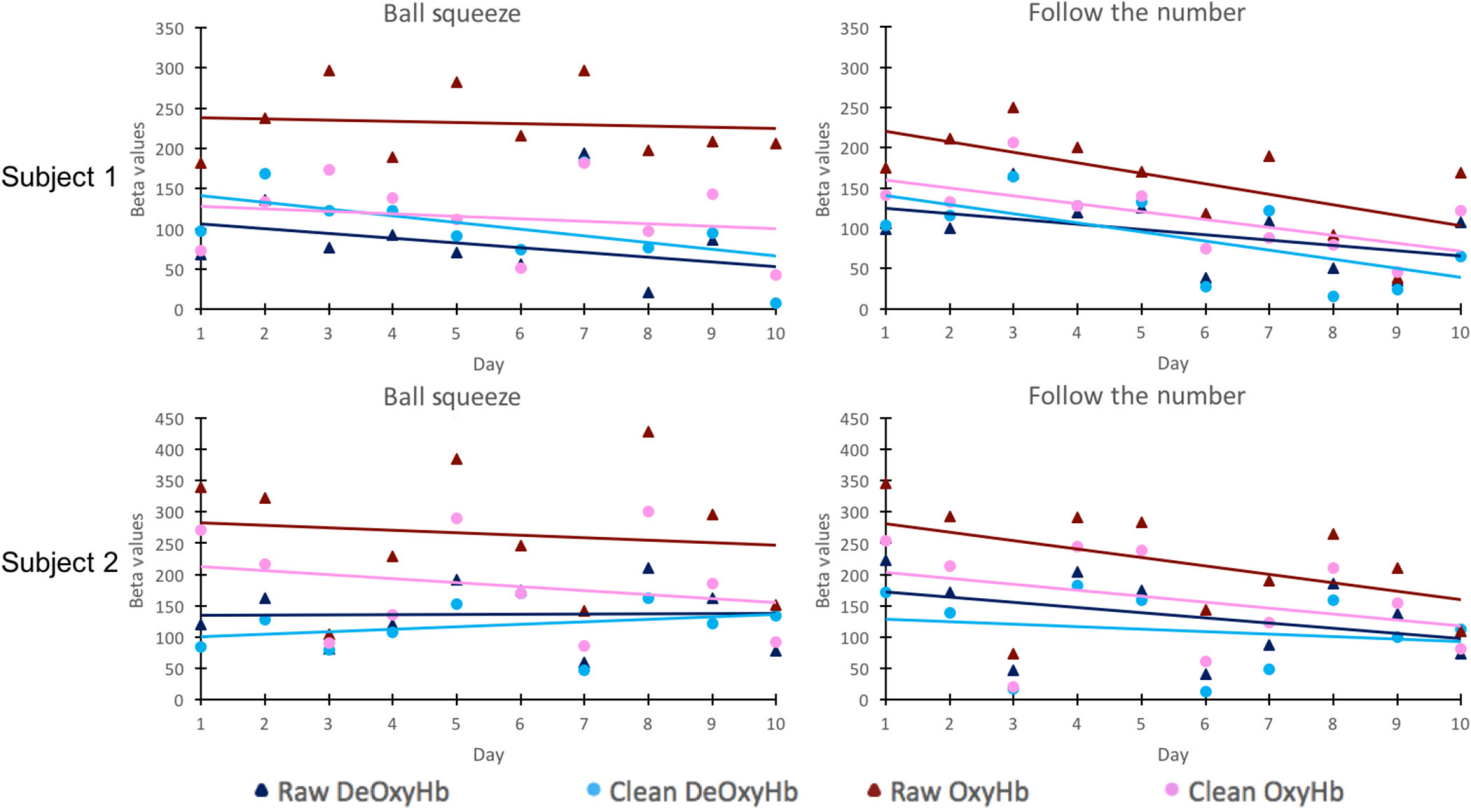
Results of 10-day longitudinal study. Top row represents data from participant 1; bottom row represents data from participant 2. Left column shows result of the ball squeeze task; right column shows result of the follow the number task. Daily beta values and trend lines are shown. Red represents OxyHb; blue represents deOxyHb. Dark colors and triangles represent raw data; lighter colors and circles represent clean data.

Both participants completed the four tasks of experiment 1 every day for 10 days. Here, we compare the results from the ball squeeze task and the follow the number task using the OxyHb and deOxyHb signals, with and without the spatial filter. The graphs in Fig. 3 show the beta values and trend lines for each signal type over the course of the 10 days for participants 1 and 2. Red points and lines represent data using the OxyHb signal and blue points and lines represent the deOxyHb signal. Triangular points and darker colors represent raw data and circular points and lighter colors represent clean (spatially filtered) data. We refer to “attenuation” over the 10 days if the slope of the line was negative and significantly different from zero (p<0.05). Slopes of each trend line are shown in Table 2. For subject 1, no signal showed attenuation over the 10 days during the ball squeeze task; although, the clean deOxyHb signals showed a negative trend (p=0.067). In the follow-the-number task, however, all signals showed attenuation (p<0.05) except the raw deOxyHb signal, which showed a trend (p=0.084). For subject 2, no signals showed attenuation, either in the ball squeeze or the follow-the-number task.

**Table 2:** Slopes of functions in Fig. 3. Asterisks (*) indicate significance (p<0.05); (−) indicates negative slope.

		DeOxyHb		OxyHb	
		Raw	Clean	Raw	Clean
Subject 1	Ball squeeze	−5.87	−8.37	−1.36	−3.09
	Follow the number	−6.58	−11.27^*^	−13.00^*^	−9.70^*^
Subject 2	Ball squeeze	0.41	3.93	−3.98	−6.31
	Follow the number	−8.31	−3.97	−13.37	−9.44

## 7 Discussion

To study the test–retest reliability of fNIRS signals, we asked participants to perform four different motor tasks on two separate days. Overall, the OxyHb signal was shown to be more reliable than the deOxyHb signal, and the reliability was also higher for the raw signal than for signals that had undergone a spatial filter. To test the extent to which these signals were stable over time, a longitudinal study was conducted in which two participants performed the same four motor tasks for 10 days. A comparison of two representative tasks with the highest and lowest test–retest reliability in the clean deOxyHb signals (follow the number and ball squeeze, respectively) showed that these tasks elicit different levels of neural and global components in some participants. For the “ball squeeze” task, which had the lowest test–retest reliability using the clean deOxyHb data, neither participant showed attenuation in any signal, OxyHb or deOxyHb, with or without the spatial filter over the course of the 10 days. However, for the “follow the number” task, which showed the highest test–retest reliability in the clean deOxyHb signal, in one participant, signals showed attenuation over 10 days, indicating that this task may elicit less systemic noise in some subjects. This study is the first to our knowledge to systematically analyze the reliability of OxyHb and deOxyHb signals with and without a spatial filter, and our findings suggest that systemic components present in fNIRS signals may be individually specific and inflate day-to-day reliability relative to the underlying neural components. Further, the global mean may inform physiological processes associated with specific functional tasks and neural mechanisms, adding insight to our understanding of neurovascular interactions.

Prior studies have shown that the OxyHb signal is more susceptible to systemic artifacts than the deOxyHb signal^12^^,^^14^^,^^15^ and our results additionally indicate that the OxyHb signal is more reliable than the deOxyHb signal. One interpretation suggests that systemic effects are more similar day to day than neural effects. Reduced reliability for the clean signals relative to the raw signals also supports this interpretation. This reliability, however, comes at the cost of functional specificity, as the activity represented by the raw OxyHb signal was widely distributed rather than limited to left motor cortex and adjacent areas. Applying a spatial filter to these results improved the functional specificity but still showed higher reliability values when compared to the deOxyHb signal, which is known to be less susceptible to systemic effects.

The group findings also support the conclusion that the deOxyHb signal, while less reliable, was less affected by systemic components than OxyHb, suggested by the more localized region of activity in Fig. 2. The reduced reliability observed for the deOxyHb signal, assumed to be primarily neural in origin, raises important questions about the nature of the interaction between systemic and neural effects between tasks and across days, and about strategies to improve signal acquisition and processing.

**Fig. 4:**
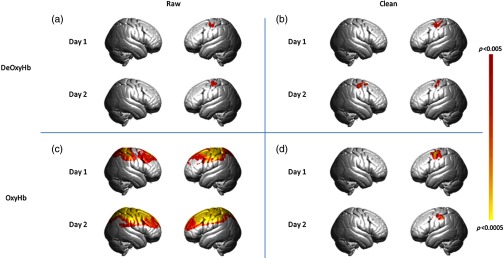
Results of ball squeeze task, p<0.005. The top row represents the group-averaged deOxyHb data; bottom row represents OxyHb data. (a) Results of raw deOxyHb data from day 1 and day 2. (b) Results of clean (spatial filter applied) deOxyHb data from day 1 and day 2. (c) Results of raw OxyHb data from day 1 and day 2. (d) Results of clean (spatial filter applied) OxyHb data from day 1 and day 2.

For the Ball squeeze task in the longitudinal experiment, the beta values for the OxyHb were higher than for the deOxyHb throughout the 10 days for all tasks in both subjects. Even when the spatial filter was applied to the OxyHb signals, the beta values remained high in both participants without evidence of attenuation. Similarly, the deOxyHb signal did not show significant attenuation for either participant during this task, even after application of the spatial filter. One interpretation of this result is that both signals obtained during this task were more influenced by systemic components. This is consistent with the group results for this task, which show widespread, non-specific activation in both hemispheres in the raw, OxyHb signals (see Appendix, Fig. 4). It is possible that the global components in the signals elicited by this task masked any putative signal attenuation over the course of the 10 days.

By contrast, the task with the highest reliability in the test–retest experiment using the clean, deOxyHb signal was follow the number, which had a cognitive component that the other tasks did not. For the follow the number task, participants were asked to move a specific finger in response to the numbered cue, requiring increased attention to execute an unpredictable sequence of finger movements. In the test–retest experiment, this task showed low reliability in the OxyHb signals, but higher reliability in the deOxyHb and especially in the clean data. This is consistent with the hypothesis that global components in the data lead to higher reliability, and this task may either elicit less systemic artifacts or the global components may be more effectively separated from neural signals in this task using our method of global mean removal. In the longitudinal experiment, for one participant, this task showed attenuation over 10 days in all signal types, supporting the hypothesis that it may not elicit as much global signal as the ball squeeze task for this subject. However, this attenuation was not present in the data from the second participant, showing that this effect was variable across participants. While the global systemic artifact increases the reliability of the data, using a task that elicits a less global signal may result in signals that are more likely to be neural in origin. However, further studies that directly record systemic measures are necessary to determine what, if any, task features cause increases or decreases in the systemic artifact accompanying neural signals.

Findings of this study suggest that day-to-day reliability of fNIRS recordings depends on both the signal and task used and that reliability may be inflated by systemic factors rather than neural activity. In general, neural and systemic components differentially affect OxyHb and deOxyHb signals. The deOxyHb signal was less reliable but the task-based effect was spatially specific. These factors are especially important in the design of pre/postintervention experiments as they influence the likelihood that changes in signals reflect neural effects of the treatment or intervention.

**Fig. 5:**
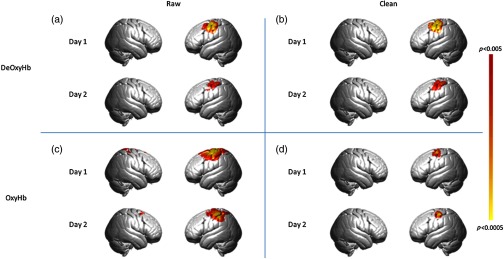
Results of double tap task, p<0.005. The top row represents the group-averaged deOxyHb data; bottom row represents OxyHb data. (a) Results of raw deOxyHb data from day 1 and day 2. (b) Results of clean (spatial filter applied) deOxyHb data from day 1 and day 2. (c) Results of raw OxyHb data from day 1 and day 2. (d) Results of clean (spatial filter applied) OxyHb data from day 1 and day 2.

**Fig. 6:**
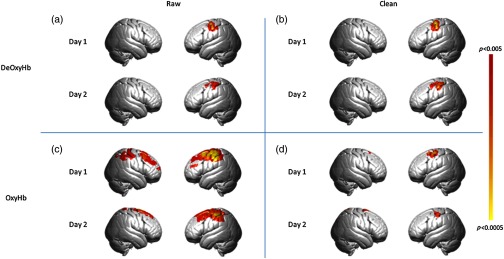
Results of single tap task, p<0.005. The top row represents the group-averaged deOxyHb data; bottom row represents OxyHb data. (a) Results of raw deOxyHb data from day 1 and day 2. (b) Results of clean (spatial filter applied) deOxyHb data from day 1 and day 2. (c) Results of raw OxyHb data from day 1 and day 2. (d) Results of clean (spatial filter applied) OxyHb data from day 1 and day 2.

## Figures and Tables

**Fig. 7:**
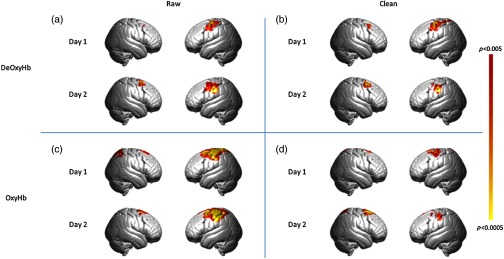
Results of follow the number task, p<0.005. The top row represents the group-averaged deOxyHb data; bottom row represents OxyHb data. (a) Results of raw deOxyHb data from day 1 and day 2. (b) Results of clean (spatial filter applied) deOxyHb data from day 1 and day 2. (c) Results of raw OxyHb data from day 1 and day 2. (d) Results of clean (spatial filter applied) OxyHb data from day 1 and day 2.

## Appendix: Group-level Task Contrast Results

Group-level results from each of the motor tasks in the test–retest experiment are shown below (Figs. 4–7). Areas in the left motor cortex from each contrast are listed in Table 3 (day one) and Table 4 (day two).

**Table 3:** Group-level GLM results from day one. Clusters of positive activity in the motor cortex and surrounding areas are listed. Horizontal lines separate results from each contrast (in bold). (−) on the x axis indicates left side. BA = Brodmann’s area. MNI coordinates were converted to Talairach coordinates to generate cluster labels.

Signal	Peak MNI coordinates	Peak T	P	# voxels	BA	Anatomical area	Probability
**Ball squeeze task**							
OxyHb, Raw	−48−18 62	6.40	0.00001	19143	3	Primary somatosensory cortex	0.31
					6	Premotor and supplementary motor cortex	0.29
					1	Primary somatosensory cortex	0.15
					2	Primary somatosensory cortex	0.13
					4	Primary motor cortex	0.10
OxyHb, clean	−42−8 64	6.66	0.00000	804	6	Premotor and supplementary motor cortex	0.79
					3	Primary somatosensory cortex	0.12
DeOxyHb, raw	−44−24 66	3.96	0.00063	202	3	Primary somatosensory cortex	0.27
					6	Premotor and supplementary motor cortex	0.25
					1	Primary somatosensory cortex	0.16
					2	Primary somatosensory cortex	0.15
					4	Primary motor cortex	0.13
DeOxyHb, clean	−44−6 62	4.65	0.00016	364	6	Premotor and supplementary motor cortex	0.80
					3	Primary somatosensory cortex	0.11
**Double tap task**							
OxyHb, raw	−40−22 68	4.87	0.00010	4802	6	Premotor and supplementary motor cortex	0.40
					3	Primary somatosensory cortex	0.25
					4	Primary motor cortex	0.17
					1	Primary somatosensory cortex	0.12
OxyHb, clean	−40−4 64	4.26	0.00034	698	6	Premotor and supplementary motor cortex	0.92
DeOxyHb, raw	−38−14 62	5.43	0.00004	1811	6	Premotor and supplementary motor cortex	0.71
					3	Primary somatosensory cortex	0.19
DeOxyHb, clean	−44−20 64	6.89	0.00000	2005	6	Premotor and supplementary motor cortex	0.35
					3	Primary somatosensory cortex	0.28
					1	Primary somatosensory cortex	0.16
					4	Primary motor cortex	0.11
**Single tap task**							
OxyHb, raw	−26−22 70	4.93	0.00009	4536	6	Premotor and supplementary motor cortex	0.58
					4	Primary motor cortex	0.27
					3	Primary somatosensory cortex	0.14
OxyHb, clean	−44−10 60	4.50	0.00021	705	6	Premotor and supplementary motor cortex	0.67
					3	Primary somatosensory cortex	0.21
DeOxyHb, Raw	−50−10 56	4.20	0.00038	661	6	Premotor and supplementary motor cortex	0.58
					3	Primary somatosensory cortex	0.21
DeOxyHb, clean	−44−18 54	4.86	0.00010	1102	3	Primary somatosensory cortex	0.36
					6	Premotor and supplementary motor cortex	0.25
					1	Primary somatosensory cortex	0.19
					4	Primary motor cortex	0.13
**Follow the number task**							
OxyHb, raw	−44−8 62	6.33	0.00001	5429	6	Premotor and supplementary motor cortex	0.74
					3	Primary somatosensory cortex	0.16
OxyHb, clean	−56−4 48	5.34	0.00004	734	6	Premotor and supplementary motor cortex	0.79
DeOxyHb, raw	−46−8 60	5.44	0.00003	846	6	Premotor and supplementary motor cortex	0.70
					3	Primary somatosensory cortex	0.20
DeOxyHb, clean	−46−8 60	5.76	0.00002	911	6	Premotor and supplementary motor cortex	0.70
					3	Primary somatosensory cortex	0.20
**All tasks**							
OxyHb, raw	−48−20 62	6.43	0.00001	14279	3	Primary somatosensory cortex	0.31
					6	Premotor and supplementary motor cortex	0.23
					1	Primary somatosensory cortex	0.18
					2	Primary somatosensory cortex	0.16
OxyHb, clean	−42−8 64	5.89	0.00001	947	6	Premotor and supplementary motor cortex	0.79
					3	Primary somatosensory cortex	0.12
DeOxyHb, raw	−46−10 60	4.98	0.00008	1375	6	Premotor and supplementary motor cortex	0.62
					3	Primary somatosensory cortex	0.22
					4	Primary motor cortex	0.10
DeOxyHb, clean	−32−6 66	6.26	0.00001	1571	6	Premotor and supplementary motor cortex	1.00

**Table 4:** Group-level GLM results from day two. Clusters of positive activity in the motor cortex and surrounding areas are listed. Horizontal lines separate results from each contrast (in bold). (−) on the x axis indicates left side. BA = Brodmann’s area. MNI coordinates were converted to Talairach coordinates to generate cluster labels.

Signal	Peak MNI coordinates	Peak T	P	# Voxels	BA	Anatomical area	Probability
**Ball squeeze task**							
OxyHb, raw	34 −42 70	6.69	0.00000	31062	5	Somatosensory association cortex	0.35
					2	Primary somatosensory cortex	0.19
					3	Primary somatosensory cortex	0.16
					7	Somatosensory association cortex	0.15
OxyHb, clean	−42 2 48	3.20	0.00296	61	6	Premotor and supplementary motor cortex	0.88
					8	Includes frontal eye fields	0.12
DeOxyHb, raw	−50−22 60	3.87	0.00075	206	3	Primary somatosensory cortex	0.31
					2	Primary somatosensory cortex	0.21
					1	Primary somatosensory cortex	0.19
					6	Premotor and supplementary motor cortex	0.11
DeOxyHb, clean	−52−22 58	4.08	0.00049	189	3	Primary somatosensory cortex	0.31
					2	Primary somatosensory cortex	0.22
					1	Primary somatosensory cortex	0.17
					40	Supramarginal gyrus part of Wernicke’s area	0.11
**Double tap task**							
OxyHb, raw	−60−20 48	4.73	0.00013	2604	6	Premotor and supplementary motor cortex	0.24
					2	Primary somatosensory cortex	0.23
					3	Primary somatosensory cortex	0.19
					1	Primary somatosensory cortex	0.17
					40	Supramarginal gyrus part of Wernicke’s area	0.11
OxyHb, clean	−54−12 52	4.74	0.00013	485	6	Premotor and supplementary motor cortex	0.52
					3	Primary somatosensory cortex	0.19
					4	Primary motor cortex	0.12
DeOxyHb, raw	−40−20 68	4.03	0.00055	548	6	Premotor and supplementary motor cortex	0.48
					3	Primary somatosensory cortex	0.24
					4	Primary motor cortex	0.16
					1	Primary somatosensory cortex	0.10
DeOxyHb, clean	−40−20 68	4.37	0.00028	786	6	Premotor and supplementary motor cortex	0.48
					3	Primary somatosensory cortex	0.24
					4	Primary motor cortex	0.16
					1	Primary somatosensory cortex	0.10
**Single tap task**							
OxyHb, raw	−50−16 58	4.51	0.00021	7521	6	Premotor and supplementary motor cortex	0.33
					3	Primary somatosensory cortex	0.28
					2	Primary somatosensory cortex	0.14
					1	Primary somatosensory cortex	0.14
					4	Primary motor cortex	0.10
OxyHb, clean	−52−14 56	4.87	0.00010	280	6	Premotor and supplementary motor cortex	0.42
					3	Primary somatosensory cortex	0.22
					1	Primary somatosensory cortex	0.13
					2	Primary somatosensory cortex	0.12
					4	Primary motor cortex	0.11
DeOxyHb, raw	−60−14 46	4.21	0.00038	440	6	Premotor and supplementary motor cortex	0.48
					3	Primary somatosensory cortex	0.19
					1	Primary somatosensory cortex	0.15
					2	Primary somatosensory cortex	0.11
DeOxyHb, clean	−60−14 46	4.63	0.00016	605	6	Premotor and supplementary motor cortex	0.48
					3	Primary somatosensory cortex	0.19
					1	Primary somatosensory cortex	0.15
					2	Primary somatosensory cortex	0.11
**Follow the number task**							
OxyHb, raw	−44−14 64	6.62	0.00000	3870	6	Premotor and supplementary motor cortex	0.56
					3	Primary somatosensory cortex	0.27
OxyHb, clean	−24−10 74	4.95	0.00009	643	6	Premotor and supplementary motor cortex	0.98
DeOxyHb, raw	−64−20 34	4.91	0.00009	1306	40	Supramarginal gyrus part of Wernicke’s area	0.26
					6	Premotor and supplementary motor cortex	0.23
					2	Primary somatosensory cortex	0.16
					1	Primary somatosensory cortex	0.14
DeOxyHb, Clean	−64−16 32	4.88	0.00010	643	6	Premotor and supplementary motor cortex	0.31
					2	Primary somatosensory cortex	0.14
					40	Supramarginal gyrus part of Wernicke’s area	0.14
					43	Subcentral area	0.13
					1	Primary somatosensory cortex	0.11
**All tasks**							
OxyHb, raw	−46−32 54	6.71	0.00000	19650	40	Supramarginal gyrus part of Wernicke’s area	0.45
					2	Primary somatosensory cortex	0.27
					1	Primary somatosensory cortex	0.18
OxyHb, clean	−52−14 56	4.75	0.00013	480	6	Premotor and supplementary motor cortex	0.42
					3	Primary somatosensory cortex	0.22
					1	Primary somatosensory cortex	0.13
					2	Primary somatosensory cortex	0.12
					4	Primary motor cortex	0.11
DeOxyHb, Raw	−60−14 46	3.87	0.00075	911	6	Premotor and supplementary motor cortex	0.48
					3	Primary somatosensory cortex	0.19
					1	Primary somatosensory cortex	0.15
					2	Primary somatosensory cortex	0.11
DeOxyHb, clean	−60−14 46	4.05	0.00052	883	6	Premotor and supplementary motor cortex	0.48
					3	Primary somatosensory cortex	0.19
					1	Primary somatosensory cortex	0.15
					2	Primary somatosensory cortex	0.11
